# Mitochondrial DNA Content Modulates Chemosensitivity and Cellular Adaptation in Glioblastoma Cells

**DOI:** 10.5152/eurasianjmed.2026.251207

**Published:** 2026-04-14

**Authors:** Siti Muslihah Ab Radzak, Azim Patar, Hsin-Chen Lee, Abdul Aziz Mohamed Yusoff

**Affiliations:** 1 Department of Neurosciences, School of Medical Sciences, Universiti Sains Malaysia, Health Campus, Kubang Kerian, Kelantan, Malaysia; 2 Department and Institute of Pharmacology, National Yang-Ming Chiao-Tung University College of Medicine, Taipei, Taiwan

**Keywords:** Glioblastoma, mitochondrial DNA copy number, mtDNA depletion, temozolomide sensitivity

## Abstract

**Background:**

Mitochondrial DNA (mtDNA) copy number alterations are closely associated with glioblastoma (GBM) progression and therapeutic resistance. This study investigates how changes in mitochondrial content affect the chemotherapeutic response of GBM cells in vitro.

**Methods:**

Glioblastoma cell lines (U87 and LN18) were initially treated with ethidium bromide (EtBr) to deplete mtDNA, followed by EtBr withdrawal to enable mtDNA restoration. Real-time Polymerase Chain Reaction (PCR) was subsequently employed to quantify mtDNA levels. To assess cellular responses, cell viability was measured using the CCK-8 assay, while wound healing and Transwell assays were conducted to evaluate cell migration and invasion. Apoptosis was analyzed via flow cytometry using the Annexin V-FITC/PI kit. In addition, intracellular reactive oxygen species (ROS) and mitochondrial membrane potential (ΔΨm) were examined by flow cytometry, and Adenosine triphosphate (ATP) levels were quantified using a luminescent ATP detection assay.

**Results:**

Glioblastoma cells with depleted mtDNA exhibited an increase in mtDNA content following temozolomide (TMZ) exposure. Despite this, these cells showed reduced viability, migration, and invasion, while also displaying adaptive responses suggestive of emerging resistance. In contrast, cells in which mtDNA content had been restored demonstrated recovery across all parameters, indicating enhanced sensitivity to TMZ.

**Conclusion:**

This study highlights the essential role of mtDNA content in modulating GBM cell sensitivity to chemotherapy, as variations in mtDNA levels can influence cellular responses to treatment. These findings suggest that mtDNA content could act as a potential biomarker and that its modulation may offer a promising strategy to overcome drug resistance in GBM.

**Main points**

- Alterations in mitochondrial DNA (mtDNA) copy number have been strongly associated with glioblastoma (GBM) progression and diminished responsiveness to chemotherapy.
- Mitochondrial DNA-depleted GBM cells exhibited elevated mtDNA levels following temozolomide (TMZ) treatment but showed reduced viability, migration, and invasion, along with adaptive changes indicative of emerging drug resistance.
- Glioblastoma cells with restored mtDNA content displayed recovered cellular functions and enhanced responsiveness to TMZ, underscoring the pivotal role of mitochondrial integrity in modulating chemotherapeutic sensitivity.

## Introduction

Glioblastoma (GBM) is the most aggressive primary brain tumor, marked by rapid growth, therapy resistance, and poor prognosis. Its global incidence ranges from 0.76 to 4.8 per 100 000 individuals annually, with GBM comprising up to 48% of primary brain tumors in the United States.^1^ Despite maximal surgery, radiotherapy, and chemotherapy, median survival remains low, with only 2% of patients surviving beyond 3 years and 0.71% reaching 10-year survival.^2^ The World Health Organization classifies GBM as a Grade 4 malignant tumor due to its high malignancy and unfavorable clinical outcomes.^1^^,^^2^

Temozolomide (TMZ), an orally bioavailable alkylating agent, is a first-line treatment for GBM.^3^ It produces an active metabolite that methylates DNA, inducing DNA damage, cell cycle arrest, and apoptosis, particularly in highly proliferative tumor cells. Temozolomide crosses the blood-brain barrier, achieving 30%-40% effective concentrations within the central nervous system (CNS).^4^ While it can delay glioma recurrence, its clinical efficacy is limited by a short half-life, chemical instability, and intrinsic or acquired resistance of GBM cells, which substantially impair long-term therapeutic outcomes.^3^

Mitochondria play a crucial role in modulating chemotherapeutic sensitivity in GBM cells. Beyond energy production, they regulate reactive oxygen species (ROS), calcium homeostasis, and apoptosis. Mitochondria contain their own 16.5 kb circular genome, mitochondrial DNA (mtDNA), which is independent of nuclear DNA and encodes essential elements for mitochondrial gene expression and replication.

Each cell contains hundreds to thousands of mtDNA copies, depending on its type and metabolic demand. Alterations in mtDNA copy number are linked to mitochondrial myopathies, neurodegenerative disorders, aging, and various cancers.^5^^,^^6^ The recent analysis revealed elevated mtDNA levels in brain tumors compared to matched blood, correlating with prolonged survival, particularly in high-grade tumors.^7^ Hence, mtDNA copy number serves as a promising diagnostic and prognostic indicator of mitochondrial health. Notably, changes in mitochondrial content occur following TMZ treatment, and mtDNA depletion is often achieved using ethidium bromide (EtBr).^8^

Low concentrations of EtBr selectively inhibit mtDNA replication without affecting the nuclear genome, allowing the study of cellular adaptations to mitochondrial dysfunction.^8^ Mitochondrial DNA depletion alters cellular responses to TMZ, affecting redox balance, apoptosis, and DNA repair.^4^ Additionally, transient mtDNA loss enhances invasiveness and apoptosis resistance, promoting tumor progression and metastasis.^9^^,^^10^

Altered mtDNA content contributes to the metabolic adaptability of cancer cells under treatment stress.^8^^,^^11^ However, the response of mtDNA-depleted GBM cells to TMZ remains unclear. This study examines how EtBr-induced mtDNA depletion affects TMZ efficacy, offering insights into mitochondrial mechanisms underlying treatment response.

## Material and Methods

All experimental procedures were conducted in the cell culture and molecular research laboratories of the Department of Neurosciences, Universiti Sains Malaysia. Ethical approval and informed consent were not required for this study, as it did not involve any human participants, human tissues, or animal models. The experiments were performed exclusively using established human-derived cell lines obtained from the American Type Culture Collection (ATCC), a recognized public cell resource. These anonymized cell lines are commercially supplied for research purposes and, in accordance with institutional regulations, do not require ethics committee approval or informed consent.

### Cell Culture and Ethidium Bromide Treatment

Human GBM cell lines (U87MG and LN18) and normal astrocytes (SVG p12) were sourced from the ATCC (Rockville, MD, USA) and cultured in Dulbecco’s Modified Eagle’s Medium with 10% FBS, 1% penicillin-streptomycin, 1% Minimum Essential Medium (MEM) non-essential amino acids, 4.5 g/L glucose, L-glutamine, and sodium pyruvate at 37°C, 5% CO_2_. To establish cells with varying mtDNA levels, GBM cells were treated with 50 ng/mL EtBr for 4 days, followed by culturing in EtBr-free medium for another 4 days to allow recovery. Total DNA was then extracted using the Geneaid Isolation Kit (Geneaid Biotech Ltd., Taiwan) and stored at –80°C.

### Determination of Half Maximal Inhibitory Concentration (IC_50_)

Cells (5 × 10^3^/well) were seeded in 96-well plates and incubated for 24 hours before treatment with 50-1000 µM TMZ (Sigma-Aldrich, St. Louis, MO, USA) for 48 hours. Cell viability was assessed using Cell Count Reagent SF (CCK-8) (Nacalai Tesque Inc., Kyoto, Japan). Absorbance at 450 nm was measured with a Tecan Sunrise microplate reader (Tecan, Zürich, Switzerland), and IC_50_ values were determined from dose-response curves.

### Determination of Mitochondrial DNA Copy Number

The relative mtDNA copy number was measured by quantitative real-time PCR (qPCR) and calculated as the ratio of the mitochondrial gene (mt-ND1) to the nuclear gene (β-actin). qPCR was performed using 2× Brilliant III SYBR Green Master Mix with Low ROX on an Mx3005P Real-Time PCR System (Agilent Technologies, Santa Clara, CA, USA). ND1 primers (5’-TCTCACCATCGCTCTTCTAC-3’; 5’-TTGGTCTCTGCTAGTGTGGA-3’) and β-actin primers (5’-CATGTGCAAGGCCGGCTTCG-3’; 5’-CTGGGTCATCTTCTCGCGGT-3’) were used as mitochondrial and nuclear targets, respectively.^12^ qPCR conditions were 95°C for 3 minutes, followed by 40 cycles of 95°C for 15 seconds and 60°C for 20 seconds. Samples were run in triplicate, and relative mtDNA content was calculated using the 2 x 2^-∆CT (CtmtDNA – CtnDNA)^ and 2^-∆∆CT^ methods.

### Cell Viability Assay

Cells with different mtDNA levels (5 × 10^3^/well) were seeded in 96-well plates, allowed to adhere for 24 hours, and treated with TMZ for 24 or 48 hours. Cell viability was then assessed using Cell Count Reagent SF (CCK-8) (Nacalai Tesque Inc., Kyoto, Japan).

### Wound Healing Assay

Cells with different mtDNA levels (1.5 × 10^5^/well) were cultured in 24-well plates for 24 hours to form monolayers. Linear scratches were made using a 100 µL pipette tip, and debris was removed with 1 × PBS. Cells were treated with TMZ in fresh medium, and scratch areas were imaged at 0, 24, and 48 hours. Wound widths were manually measured using ImageJ software (v1.52a, U.S. National Institutes of Health, Bethesda, Maryland, USA) to evaluate cell migration.

### Migration and Invasion Assay

Cell invasion was assessed using Transwell inserts with 8-µm pore membranes (Nest Scientific Inc., Rahway, NJ, USA). The membranes were initially pre-coated with Geltrex basement membrane matrix (Thermo Fisher Scientific, Waltham, MA, USA) and incubated at 37°C for 1 hour. Serum-starved U87 (3 × 10^4^) and LN18 (1 × 10^5^) cells were seeded into the upper chamber, with medium containing 10% FBS in the lower chamber. After 24 hours, migrated or invaded cells were fixed with methanol, stained with crystal violet, and quantified microscopically.

### Mitochondrial Membrane Potential Assay

Mitochondrial membrane potential (Δψm) was assessed using the JC-1 fluorescent probe (Elabscience, Wuhan, Hubei, China) according to the manufacturer’s protocol. Treated cells (1 × 10^8^) were incubated with JC-1 at 37°C for 20 minutes and analyzed using a BD FACSCanto II Flow Cytometer (BD Biosciences, San Jose, CA, USA), where reduced red-to-green ratios indicated mitochondrial depolarization.

### Measurement of Cellular ATP Levels

ATP production was measured in cells with different mtDNA levels (1.2 × 10^4^/well) cultured in 96-well white plates and treated with TMZ for 24 hours. Levels were quantified using the Luminescent ATP Detection Assay Kit (Abcam, Cambridge, MA, USA) and read on a SpectraMax M5 Microplate Reader (Molecular Devices, Sunnyvale, CA, USA).

### Intracellular Reactive Oxygen Species Assay

Intracellular ROS levels were assessed using the 2’,7’-dichlorodihydrofluorescein diacetate (DCFH-DA) fluorescent probe (Elabscience, Wuhan, Hubei, China). Treated cells were incubated with 20 µM probe at 37°C for 45 minutes, washed, and analyzed by BD FACSCanto II Flow Cytometry (BD Biosciences, San Jose, CA, USA). Fluorescence intensity was quantified using FlowJo v10.9 (FlowJo LLC, Ashland, OR, USA).

### Apoptosis Assay

Apoptotic cell death was evaluated using the Annexin V-FITC/PI Apoptosis Kit (Elabscience, Wuhan, Hubei, China). Treated cells were harvested, stained with Annexin V-FITC and PI, and analyzed with a BD FACSCanto II Flow Cytometer (BD Biosciences, San Jose, CA, USA) to distinguish viable, early apoptotic, and late apoptotic or necrotic cells.

### Statistical Analysis

Data were analyzed using SPSS v27.0 (IBM Corp., Armonk, NY, USA). Mitochondrial DNA content differences were assessed by Kruskal-Wallis, and treatment effects by 1-way analysis of variance (ANOVA) with Dunnett’s post hoc test. Wound healing progression was evaluated using repeated measures ANOVA. Results are presented as mean ± SD, with significance at *P* < 0.05.

## Results

### Higher Half Maximal Inhibitory Concentration in Glioblastoma Cells

**Figure 1.**
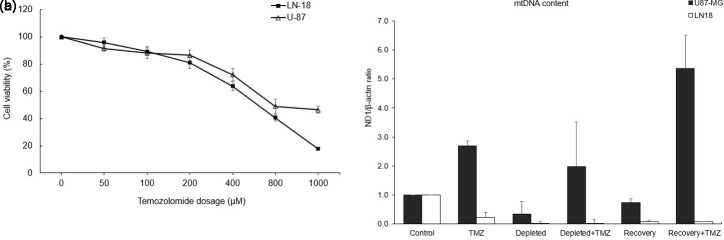
Effects of ethidium bromide (EtBr) and temozolomide (TMZ) on mitochondrial DNA (mtDNA) content in glioblastoma (GBM) cell lines. A. Determination of the half-maximal inhibitory concentration (IC_50_) for U87 and LN18 cell lines. Dose-response curves indicated a higher IC_50_ for TMZ in U87 cells (930 µM) compared to LN18 cells (620 µM). B. Comparison of mtDNA content between normal astrocytes and GBM cell lines, and the effect of TMZ on mtDNA levels in EtBr-induced GBM cells. Low-dose EtBr treatment reduced mtDNA content in U87 and LN18 cells, whereas TMZ treatment resulted in an increase in mtDNA levels in mtDNA-depleted U87 and LN18 cells. Mitochondrial DNA depletion was induced by treating cells with EtBr for 4 days, followed by a 4-day recovery period in EtBr-free medium. Relative mtDNA levels were quantified by real-time PCR and normalized to those of normal astrocyte cell lines. Data are presented as mean ± SD (n = 3) (***P < .001 vs. control).

In this study, the cytotoxicity of TMZ in GBM cells was evaluated using a CCK-8 assay after 48 hours. U87 cells exhibited a higher IC_50_ (930 µM) than LN18 cells (620 µM), indicating lower sensitivity to TMZ, likely due to inherent molecular differences. These IC_50_ values were used as standardized doses for subsequent experiments (Figure 1A).

### Restoration of Mitochondrial DNA Content in Mitochondrial DNA-Depleted Glioblastoma Cells After Temozolomide Treatment

Mitochondrial DNA depletion in GBM cell lines was successfully induced by treating the cells with EtBr for 4 days, as it intercalates between mtDNA base pairs. Lacking protective histones, mtDNA is structurally disrupted by EtBr, which impairs replication and transcription and progressively reduces mtDNA content over time.

Mitochondrial DNA depletion was confirmed by qPCR using mtDNA-specific primers, with nuclear genes serving as internal controls. Previous studies have shown that prolonged low-dose treatment with EtBr selectively depletes mtDNA, and this effect is reversible upon EtBr withdrawal.^13^ In contrast, nuclear DNA, protected by histones and robust repair mechanisms, is minimally affected, with nuclear gene levels remaining consistent between parental and EtBr-treated cells, indicating selective mtDNA depletion without disrupting nuclear DNA replication.

To examine the impact of mtDNA depletion on drug sensitivity, LN18 and U87 cells were co-treated with EtBr and TMZ, and mtDNA levels also measured in normal astrocytes. Parental GBM cells exhibited higher mtDNA content than astrocytes. Temozolomide treatment altered mitochondrial content, reducing mtDNA in LN18 cells while increasing it in U87 cells. Interestingly, TMZ also elevated mtDNA levels in mtDNA-depleted cells of both lines, suggesting an adaptive restoration mechanism to maintain mitochondrial function under chemotherapy-induced stress. These findings indicate that TMZ modulates mtDNA differently across GBM cell lines, reflecting distinct cellular responses to treatment (Figure 1B).

### Reduced Mitochondrial DNA Content Diminishes Cell Viability in Glioblastoma Cells

**Figure 2.**
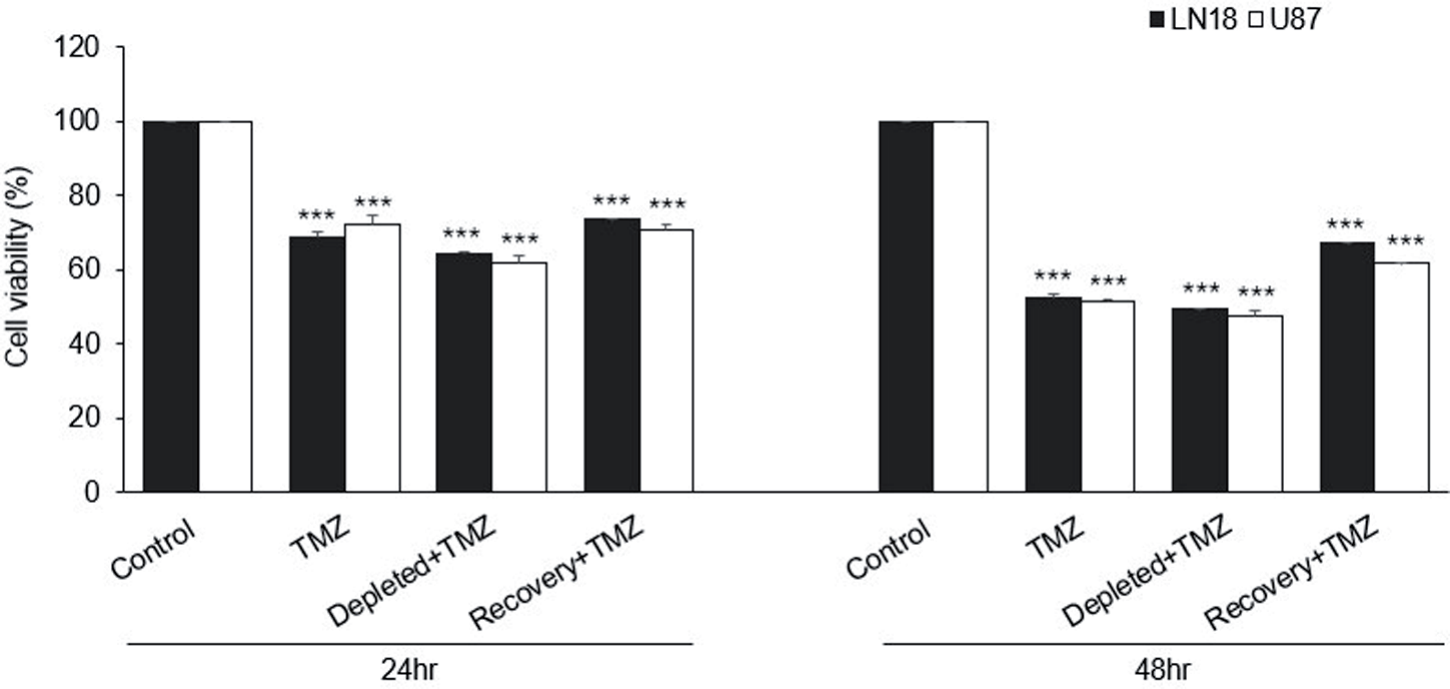
Cell viability assay of glioblastoma (GBM) cell groups following temozolomide (TMZ) treatment. The cell viability assay demonstrated that TMZ treatment reduced cell viability across all GBM cell groups. Both parental LN18 and U87 cells exhibited decreased viability compared to their respective untreated controls, with a more pronounced reduction observed in the mitochondrial DNA (mtDNA)-depleted groups. Restoration of mtDNA content in the recovery-phase LN18 and U87 cells significantly improved cell viability. Data are presented as mean ± SD (n = 3) (*** *P* < .001 vs. control).

The functional impact of mtDNA depletion on GBM cell viability was assessed using the CCK-8 assay in U87 and LN18 cells treated with TMZ (Figure 2). Temozolomide reduced viability in both U87 (72.0% ± 2.54%) and LN18 (68.7% ± 1.72%) cells, with greater reduction in mtDNA-depleted cells (U87: 62.1% ± 1.61%; LN18: 64.5% ± 0.50%). Cells with restored mtDNA showed increased viability (U87: 70.6% ± 1.46%; LN18: 73.6% ± 0.26%), indicating a potential compensatory mechanism following TMZ exposure.

At 48 hours, TMZ-treated parental cells showed reduced viability (LN18: 53% ± 0.72%; U87: 52% ± 0.39%), with mtDNA-depleted cells exhibiting further decline (LN18: 49.3% ± 0.05%; U87: 47.3% ± 1.84%). In contrast, cells with restored mtDNA maintained higher viability (LN18: 67.2% ± 0.21%; U87: 61.6% ± 0.29%), emphasizing the critical role of mtDNA in regulating drug response and cell survival.

### Temozolomide Inhibits Migration and Invasion of Glioblastoma Cells with Depleted Mitochondrial DNA Content

**Figure 3.**
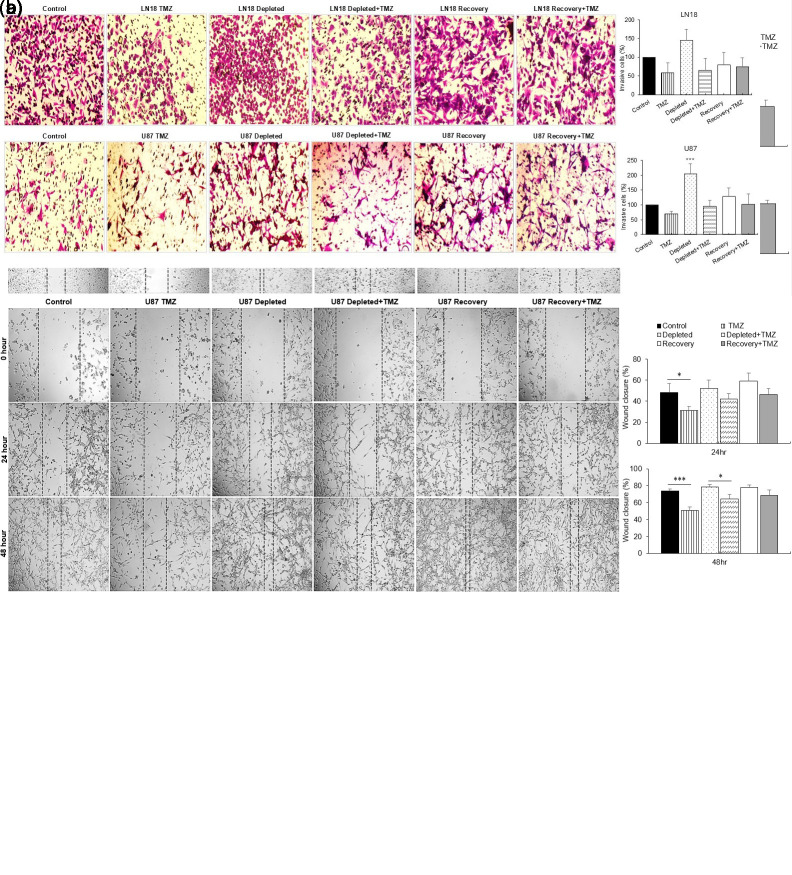
Effects of temozolomide (TMZ) therapy on cell migration and invasion in glioblastoma (GBM) cells with varying mitochondrial DNA (mtDNA) content. A) Representative images and bar graph of the scratch wound healing assay in LN18 and U87 cells. Scratch areas were monitored at 0 hour, 24 hours, and 48 hours post-treatment under 100× magnification. The percentage of wound closure was analyzed using ImageJ software. B) Invasion assays in GBM cells were conducted using Transwell inserts following 24 hours of TMZ exposure. Cells that migrated or invaded into the lower chamber were counted under 100× magnification. The number of migrating and invading cells in the control group was set at 100%. Data are presented as mean ± SD (n = 2) (*** *P* < .001 vs. control).

Highly invasive cells drive aggressive GBM progression, and mitochondrial dysfunction can impair energy-dependent processes such as cell motility. To assess this, a wound healing assay was performed on mtDNA-depleted U87 and LN18 cells following TMZ exposure (Figure 3A). After 24 hours under untreated conditions, wound closure increased from parental cells (LN18: 33.4% ± 3.97%; U87: 48.5% ± 8.17%) to mtDNA-depleted cells (LN18: 39.9% ± 3.23%; U87: 52.1% ± 8.08%) and recovery cells (LN18: 24.8% ± 5.40%; U87: 59.3% ± 7.36%). By 48 hours, closure further increased: parental (LN18: 64.0% ± 1.87%; U87: 73.7% ± 2.41%), depleted (LN18: 82.9% ± 0.57%; U87: 78.5% ± 2.99%), and recovery cells (LN18: 74.0% ± 9.17%; U87: 77.6% ± 3.02%), highlighting the influence of mtDNA content on migratory capacity.

Temozolomide treatment significantly impaired migration in parental GBM cells, reducing wound closure to 28.3% ± 5.90% in LN18 and 31.1% ± 3.90% in U87, indicating suppression of metastatic behavior. Mitochondrial DNA-depleted cells also showed reduced migration (LN18: 25.5% ± 1.40%; U87: 42.2% ± 5.07%). In contrast, mtDNA-recovered U87 cells exhibited modestly increased migration (46.5% ± 5.38%), while recovered LN18 cells remained relatively stable (24.2% ± 3.92%), suggesting limited restoration of motility in LN18.

At 48 hours, TMZ-treated U87 and LN18 cells showed further wound closure, indicating sustained migratory activity. Despite overall increases over time, the relative differences between parental, mtDNA-depleted, and mtDNA-recovered groups remained consistent, highlighting the persistent impact of mtDNA status on cell migration under TMZ treatment.

Transwell invasion assays (Figure 3B) confirmed that TMZ significantly reduced the migratory and invasive capacities of U87 and LN18 cells. Mitochondrial DNA-depleted cells treated with TMZ exhibited marked decreases in both behaviors, while mtDNA-recovered cells gradually regained migratory and invasive activity, consistent with wound healing assay results.

### Temozolomide-Induced Alterations in Mitochondrial Function in Mitochondrial DNA-Depleted Glioblastoma Cells

**Figure 4.**
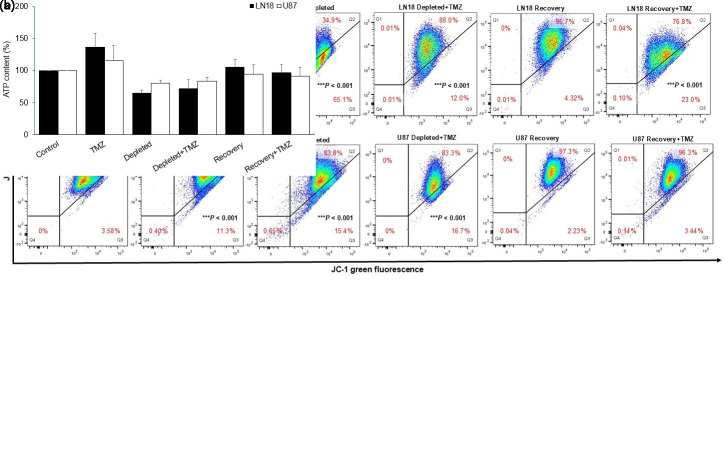
Effects of temozolomide (TMZ) treatment on mitochondrial function in glioblastoma cells with varying mitochondrial DNA content. A) Changes in mitochondrial membrane potential (ΔΨm) were assessed using JC-1 dye and analyzed by flow cytometry following 24 hours of TMZ treatment. A loss of mitochondrial ΔΨm was indicated by a shift to green fluorescence. Representative data are shown for LN18 and U87 cells (n = 3). B) Intracellular ATP levels were measured by a luminescence detection assay and normalized to the control group (set at 100%) 24 hours after TMZ treatment. ATP levels in LN18 and U87 cells are presented, illustrating the differences among the experimental groups (n = 5). Data are expressed as mean ± SD (*** *P* < .001 vs. control).

Mitochondrial Δψm is a key indicator of oxidative phosphorylation and cellular energy status.^14^ Since mtDNA-depleted cells are expected to have impaired respiration, Δψm and ATP production following TMZ treatment were assessed. JC-1 staining revealed distinct Δψm loss in TMZ-treated LN18 and U87 cells, indicating differential mitochondrial dysfunction between the 2 lines (Figure 4A).

Under untreated conditions, LN18 cells exhibited minimal mitochondrial Δψm depolarization (1.81% ± 0.70%). Ethidium bromide-induced mtDNA depletion caused substantial Δψm loss (65.13% ± 2.45%), whereas mtDNA-recovered cells showed improved mitochondrial function, with depolarization reduced to 4.32% ± 1.30%. Following TMZ treatment, LN18 cells showed marked mitochondrial Δψm loss (61.9% ± 1.10%). Mitochondrial DNA-depleted cells exhibited hyperpolarization with minimal depolarization (12.0% ± 2.10%), suggesting an adaptive response, while mtDNA-recovered cells showed a moderate Δψm decline (23.0% ± 3.91%), indicating partial restoration of mitochondrial function.

In U87 cells, mitochondrial Δψm remained largely intact. Untreated cells showed minimal depolarization (3.58% ± 1.88%), while mtDNA-depleted cells exhibited mild depolarization (15.4% ± 1.60%), indicating limited EtBr impact. Mitochondrial membrane potential in recovery cells remained stable, comparable to untreated cells, suggesting effective restoration of mitochondrial function.

Following TMZ treatment, U87 cells exhibited mild mitochondrial depolarization (11.3% ± 1.60%), indicating a less responsive phenotype. Mitochondrial DNA-depleted cells treated with TMZ showed similar depolarization (16.7% ± 1.35%), suggesting no further mitochondrial impairment, while mtDNA-recovered cells displayed a slight reduction in depolarization (3.44% ± 0.64%).

Disruption of mitochondrial Δψm negatively impacted ATP production in both GBM cell lines, with ATP levels reflecting Δψm changes. The reduction was more pronounced in LN18 cells, consistent with their greater mitochondrial depolarization (Figure 4B).

**Figure 5.**
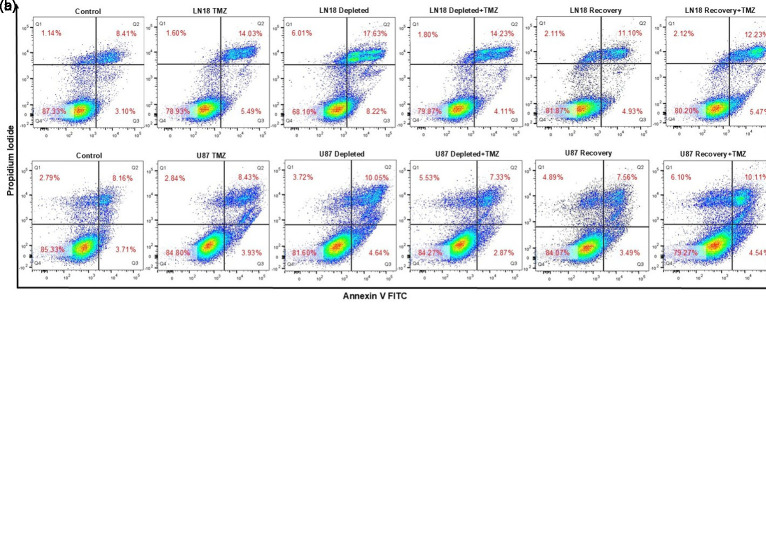
Detection of reactive oxygen species (ROS) and apoptosis in glioblastoma cells with varying mitochondrial DNA content following 24-hour temozolomide (TMZ) treatment. Reactive oxygen species levels were quantified using the DCFH-DA fluorescence probe, while apoptosis was evaluated using Annexin V-FITC/PI staining and analyzed by flow cytometry. A) The ROS levels in LN18 and U87 cell groups are illustrated, with the red line indicating untreated controls and the green line representing TMZ-treated cells. The mean fluorescence intensity of each group was quantified relative to the control group, which was set at 100% (n = 3). B) The numbers in each quadrant indicate the percentages of viable (Q4: Annexin⁻/PI⁻), early apoptotic (Q3: Annexin⁺/PI⁻), late apoptotic (Q2: Annexin⁺/PI⁺), and necrotic (Q1: Annexin⁻/PI⁺) cells, respectively. The total apoptotic cells were calculated by combining the percentages from the lower right (early apoptotic, Q3) and upper right (late apoptotic, Q2) quadrants. Data are presented as mean ± SD values from 3 independent experiments (n = 3).

Reactive oxygen species regulate both cell survival and death, with elevated ROS damaging mtDNA and mitochondrial function, creating a cycle that promotes apoptosis and chemotherapy resistance.^15^ To assess mtDNA depletion effects under TMZ, ROS levels were measured in GBM cells. Mitochondrial DNA-depleted LN18 and U87 cells showed increased ROS (LN18: 111.2% ± 20.54%; U87: 116.2% ± 19.39%). In recovery groups, ROS further increased in LN18 cells (136.2% ± 24.17%) but decreased in U87 cells (92.5% ± 8.50%), highlighting differential oxidative responses based on mtDNA content (Figure 5A).

Temozolomide treatment increased ROS levels in LN18 cells (110% ± 19.31%), indicating oxidative stress, whereas ROS decreased in U87 cells (91.4% ± 15.66%), suggesting differential TMZ responses. Mitochondrial DNA-depleted cells showed reduced ROS (LN18: 93.9% ± 8.52%; U87: 88.5% ± 8.12%), indicating protective mechanisms, while recovery-phase cells exhibited significant ROS elevation (LN18: 149.0% ± 25.54%; U87: 111.8% ± 10.98%) post treatment.

Elevated ROS levels corresponded with increased apoptosis in TMZ-treated GBM cells (Figure 5B). Mitochondrial DNA-depleted LN18 and U87 cells showed higher apoptosis (LN18: 25.9% ± 3.63%; U87: 14.7% ± 2.39%) vs. untreated controls. Recovery-phase cells exhibited reduced apoptosis (LN18: 16.0% ± 3.46%; U87: 11.0% ± 1.09%). Temozolomide increased apoptosis in parental cells (LN18: 19.5% ± 2.95%; U87: 12.4% ± 1.01%), whereas mtDNA-depleted cells showed lower rates (LN18: 18.3% ± 2.10%; U87: 10.2% ± 0.76%). Mitochondrial DNA-recovered LN18 cells maintained consistent apoptosis (17.7% ± 3.60%), while U87 recovery cells showed increased apoptosis (14.6% ± 2.01%) post treatment.

## Discussion

Theoretically, tumor cells are anticipated to proliferate uncontrollably, accompanied by elevated mtDNA levels to sustain their survival. However, persistent mtDNA depletion has been correlated with impaired replication capacity and suppressed cellular proliferation.^16^ Moreover, reduced mtDNA content has been associated with decreased sensitivity to chemotherapy.^8^^,^^11^ Therefore, this study sought to examine how variations in mtDNA levels influence the responsiveness of GBM cells to chemotherapeutic agents.

To establish a dependable baseline, astrocytes, representing the non-malignant cellular population of the CNS, serve as a crucial control for comparing mtDNA content with that of the U87 and LN18 GBM cell lines. The present findings revealed that astrocyte cells (SVG p12) contain lower mtDNA levels than the parental GBM lines LN18 and U87, possibly indicating tighter regulation of mitochondrial biogenesis and a more balanced energy requirement. Furthermore, embryonic stem cells are known to sustain minimal mtDNA copy numbers, with cell-specific increases occurring during differentiation to satisfy the metabolic needs of specialized cells.^16^^,^^17^ Hence, deviations from optimal mtDNA thresholds may prove harmful and have been associated with several pathological conditions.^18^

In this study, U87 and LN18 cells exhibited distinct alterations in mtDNA content following TMZ treatment, indicating that the response is governed by cell type-specific regulatory mechanisms. Notably, both the parental and recovery groups of LN18 cells displayed a reduction in mtDNA levels after TMZ exposure, suggesting that LN18 cells are more susceptible to the chemotherapeutic treatment than U87 cells. These observations correspond with earlier reports showing that LN18 cells possess greater sensitivity to TMZ compared with U87 cells.^19^ Additionally, TMZ has been reported to elevate mtDNA copy number in SHG44 GBM cells, consistent with the increased mtDNA levels observed in U87 cells.^20^ In the present study, the IC_50_ value of TMZ was substantially higher in U87 cells than in LN18 cells, indicating reduced drug responsiveness or intrinsic resistance. Significantly, U87 cells are characterized by extensive genetic alterations and pronounced tumor heterogeneity, factors that likely contribute to their aggressive phenotype and diminished therapeutic response.^21^ Previous research has also suggested that low mtDNA content enhances tumor susceptibility to chemotherapeutic agents, whereas elevated mtDNA levels may serve as a defensive mechanism, facilitating resistance to apoptosis.^22^

Furthermore, a substantial reduction in mtDNA content was successfully induced in both GBM cell lines following EtBr treatment. Interestingly, subsequent exposure to TMZ led to an elevation in mtDNA levels in EtBr-depleted U87 and LN18 cells. These observations suggest that treatment-induced acute stress may regulate mtDNA biogenesis as part of a cellular defense or compensatory response.^23^ Notably, an increase in mtDNA content has previously been linked to an adaptive mechanism aimed at preserving mitochondrial function.^23^ However, it has been proposed that chemically induced mtDNA depletion does not produce a uniform reduction across all cells within a population.^24^ In this regard, although the overall mtDNA levels in U87 and LN18 cells were reduced, a subset of cells may have retained relatively higher mtDNA content, potentially conferring a survival advantage under stress conditions such as chemotherapy. Moreover, TMZ has been reported to trigger metabolic reprogramming,^25^ suggesting that the observed rise in mtDNA levels following EtBr exposure may reflect an attempt to restore mitochondrial function. Therefore, a persistent increase in mtDNA content after treatment may not necessarily represent cellular restoration but rather a survival strategy that promotes tumor adaptability.

Temozolomide has been proposed to drive differentiated GBM cells toward reacquiring features characteristic of cancer stem-like cells, including self-renewal potential, chemoresistance, and heightened tumorigenic capacity, which are often correlated with increased mtDNA levels.^26^^,^^27^ Furthermore, several studies have reported that mtDNA depletion induced by EtBr renders GBM cells more resistant to TMZ, accompanied by a marked reduction in their proliferation rate.^8^^,^^11^^,^^28^ In agreement with these reports, the present study demonstrated a significant decline in cell viability in mtDNA-depleted cells after TMZ exposure, paradoxically suggesting a resistant phenotype. Notably, while these cells exhibited aggressive migratory and invasive behaviors under normal conditions, their motility and invasiveness diminished under TMZ-induced stress. Comparable findings have shown that cancer cells exhibit reduced proliferation, migration, and invasion following TMZ treatment.^29^^,^^30^ These characteristics may represent a shift toward a more quiescent, stem-like state, enabling therapeutic evasion and promoting tumor persistence.

In this study, TMZ treatment induced significant mitochondrial membrane depolarization in GBM cells, with the effect being most pronounced in the LN18 cell line. Consistent with this observation, He et al^31^ reported a moderate reduction in mitochondrial Δψm in U87 (17.48%) and U251 (16.6%) cells, as determined by JC-1 staining. Furthermore, several studies have shown that TMZ activates autophagy in glioma cells, which may transiently enhance ATP production.^32^^,^^33^ Notably, the findings revealed that GBM cells with markedly depleted mtDNA initially exhibited reduced Δψm and ATP levels; however, both parameters increased following TMZ exposure, suggesting a potential metabolic adaptation involving upregulated Oxidative phosphorylation (OXPHOS) to counter cellular stress. Supporting this, He et al^31^ demonstrated that TMZ-resistant glioma cells preserve mitochondrial membrane stability by minimizing depolarization, whereas Oliva et al^11^ observed that cells with reduced mtDNA copy numbers displayed suppressed glycolytic activity accompanied by increased ATP synthesis.

In the present study, although JC-1 dye was utilized to assess mitochondrial Δψm, potential interference from the red fluorescence of EtBr might have influenced the accuracy of the measurements in GBM cells. Nonetheless, a distinct difference in Δψm responses between treated and untreated groups was evident, suggesting that the findings were reliable and representative of the treatment’s impact. Supporting this, earlier studies have employed a comparable method in which EtBr was used to generate Rho0 cells (mtDNA-deficient), followed by evaluation of mitochondrial Δψm using the JC-1 assay.^10^

Furthermore, the results demonstrated that TMZ treatment elevated ROS levels and triggered apoptosis in LN18 cells, underscoring its crucial role in promoting cell death and suppressing tumor progression. In contrast, a slight decline in ROS levels was detected in U87 cells, possibly attributable to their intrinsic heterogeneity and variable drug sensitivity. This finding is consistent with previous studies reporting unaltered ROS generation in U87 cells following TMZ exposure.^20^ Conversely, the reduction in ROS levels observed in mtDNA-depleted cells after TMZ treatment was accompanied by a lower rate of apoptosis compared with untreated mtDNA-depleted cells. Similarly, Chen et al^34^ documented decreased ROS production and diminished apoptosis in mtDNA-deficient human osteosarcoma cell lines, indicating a relationship between mtDNA content, ROS generation, and apoptotic activity. More recently, TMZ-resistant GBM cells have been shown to exhibit reduced proliferative potential and limited apoptosis following treatment.^28^

It is well established that cancer cells frequently undergo metabolic reprogramming, transitioning between mitochondrial OXPHOS and aerobic glycolysis in accordance with their energetic demands and surrounding conditions. Collectively, the present findings suggest the activation of a cellular survival mechanism that enables resistance to therapeutic stress. Specifically, cells with depleted mtDNA appear to endure by limiting oxidative damage, depending on altered mitochondrial function to produce reduced levels of ROS while maintaining adequate ATP synthesis. As a result, this metabolic adaptation seems to diminish their susceptibility to apoptosis.

Alternatively, the restoration of mtDNA content appears to reverse the resistant phenotype previously observed in mtDNA-depleted cells. This reversal is characterized by renewed responsiveness to chemotherapy, as reflected by increased cellular sensitivity across all evaluated parameters. The results demonstrated that replenishing mtDNA content heightened the susceptibility of GBM cells to TMZ treatment, with their response closely resembling that of the parental cell lines. In agreement with this, several reports have shown comparable expression profiles between mtDNA-restored cells and untreated controls.^13^^,^^16^ Furthermore, earlier studies have proposed that cellular mtDNA levels directly modulate apoptotic sensitivity, with cells possessing higher mtDNA content exhibiting a greater propensity for programmed cell death.^35^ Hence, restoring mitochondrial content may reestablish the mitochondrial signaling required to initiate apoptosis, thereby augmenting the efficacy of chemotherapy.

In summary, these findings highlight the critical role of mtDNA depletion in shaping the functional and metabolic mechanisms involved in GBM cells, particularly in response to chemotherapeutic stress. The metabolic shift observed in highly malignant cells toward glycolytic compensation and survival adaptability underscores the importance of mitochondria in tumor progression. Therefore, further investigations are necessary to clarify the underlying mechanisms connecting mitochondrial integrity with therapy resistance in GBM and to develop strategies to overcome chemoresistance.

## Data Availability

The data that support the findings of this study are available on request from the corresponding author.
